# Audit identified modifiable factors in Hospital Care of Newborns in low-middle income countries: a scoping review

**DOI:** 10.1186/s12887-021-02965-w

**Published:** 2022-02-18

**Authors:** Muthoni Ogola, Emily Mbaire Njuguna, Jalemba Aluvaala, Mike English, Grace Irimu

**Affiliations:** 1 Health Services Unit, KEMRI-Wellcome Trust Research Programme, 197 Lenana Place, Lenana Road, P. O. Box 43640, Nairobi, 00100 Kenya; 2 Paediatrics and Child Health, University of Nairobi, Nairobi, Kenya; 3 Pumwani Maternity Hospital, Nairobi, Kenya; 4 Centre for Tropical Medicine and Global Health, Nuffield Department of Medicine, University of Oxford, Oxford, UK

**Keywords:** Newborn, Perinatal, Maternal, Mortality, Clinical, Audit process, Modifiable factors

## Abstract

**Background:**

Audit of facility-based care provided to small and sick newborns is a quality improvement initiative that helps to identify the modifiable gaps in newborn care (BMC Pregnancy Childbirth 14: 280, 2014). The aim of this work was to identify literature on modifiable factors in the care of newborns in the newborn units in health facilities in low-middle-income countries (LMICs). We also set out to design a measure of the quality of the perinatal and newborn audit process.

**Methods:**

The scoping review was conducted using the methodology outlined by Arksey and O’Malley and refined by Levac et al, (Implement Sci 5:1-9, 2010). We reported our results using the PRISMA Extension for Scoping Reviews (PRISMA-ScR) guidelines. We identified seven factors to ensure a successful audit process based on World Health Organisation (WHO) recommendations which we subsequently used to develop a quality of audit process score.

**Data sources:**

We conducted a structured search using PubMed, CINAHL, EMBASE, LILACS, POPLINE and African Index Medicus.

**Study selection:**

Studies published in English between 1965 and December 2019 focusing on the identification of modifiable factors through clinical or mortality audits in newborn care in health facilities from LMICs.

**Data extraction:**

We extracted data on the study characteristics, modifiable factors and quality of audit process indicators.

**Results:**

A total of six articles met the inclusion criteria. Of these, four were mortality audit studies and two were clinical audit studies that we used to assess the quality of the audit process. None of the studies were well conducted, two were moderately well conducted, and four were poorly conducted. The modifiable factors were divided into three time periods along the continuum of newborn care. The period of newborn unit care had the highest number of modifiable factors, and in each period, the health worker related modifiable factors were the most dominant.

**Conclusion:**

Based on the significant number of modifiable factors in the newborn unit, a neonatal audit tool is essential to act as a structured guide for auditing newborn unit care in LMICs. The quality of audit process guide is a useful method of ensuring high quality audits in health facilities.

**Supplementary Information:**

The online version contains supplementary material available at 10.1186/s12887-021-02965-w.

## Background

The newborn survival gap between high-income countries (HICs) and lower and middle income countries (LMICs) has widened over the past few decades with sub-Saharan Africa (SSA) accounting for the highest neonatal mortality rate (NMR) globally [1]. Majority of the neonatal deaths in SSA are from preventable causes which can be mitigated through basic and affordable interventions [1, 2]. Lessons can be borrowed from HICs which reduced their NMR from ≥40/1000 to < 15/1000 live births between 1900 to 1960s by optimising basic neonatal care interventions. Only after this did the provision of intensive care services in the newborn units enable a further reduction in the NMR [3]. This is evidence that the high NMR in SSA is not inevitable despite the limited resources [4].

Newborn mortality is a complex problem involving multiple factors and requiring multiple interrelated and simultaneous strategies to effectively address it [5]. Over the years, several interventions have been put in place with an aim to improve newborn care in LMICs [6]. Some of these interventions include the use of antenatal corticosteroids to stimulate lung maturity in preterm births, promotion of warmth, hygiene and exclusive breastfeeding for the preterm newborn through kangaroo mother care (KMC), prevention of mother to child transmission of HIV and training of health workers on newborn resuscitation [7–10]. Many of these strategies are however put in place to address specific challenges in isolation rather than applying a holistic approach that acknowledges the complexity of neonatal care and of health systems in general [11]. An effective strategy to reduce neonatal mortality is one that understands the linkages, interactions and feedback between the different elements of the health system [5].

Quality of care audits are a process of conducting a systematic review of patient management and comparing care provided against the accepted standards of care [12]. These can be:

i. Mortality audits which refer to the process of capturing information on the number and causes of neonatal deaths and conducting a systematic analysis of the quality of care that was provided to these newborns [12].
ii. Clinical audits which are audits on newborns who suffer a life-threatening condition following birth, however, survive the first 28 days of life [13].

The audit process uses the root cause analysis method to identify the underlying modifiable deficiencies in the barriers that have been put in place within the health system to protect the patient from harm [14, 15]. Modifiable factors or deficiencies are defined as “factors which if done differently could have prevented an adverse event from occurring” [12]. Adverse events are defined as “unintended harm that results in temporary or permanent disability, death or prolonged hospital stay” [16]. The root cause analysis method recognises that errors in patient care commonly occur due to gaps within the health system [15]. Ferlie and Shortell (2000) developed a model where the healthcare system is divided into four “nested” levels that include: i) individual patients ii) health care providers iii) infrastructure and resources and iv) environment [17]. The modifiable factors can be categorised based on this model to pinpoint where exactly in the health system action is required. Due to the interactions between different elements of the health system, when the modifiable gap is identified and acted on, it causes a ripple effect that affects other aspects of the system [5]. This is evident with the success of the maternal and perinatal audit process such as the perinatal problem identification programme (PPIP) in South Africa [18]. For the audit process to lead to improved quality in newborn care, certain factors should be put in place such as those recommended by the World Health Organisation (WHO). These include; a favourable environment, presence of an audit committee, multidisciplinary attendance of audit meetings and completion of the six step audit cycle which includes: Identification of cases, collecting information, identifying the cause of death and modifiable factors, recommending solutions, implementing recommended solutions and monitoring and evaluation [12]. Designing a process measure to assess the quality of the newborn audit process may be useful to assess and encourage the adherence of health workers to WHO guidelines on conducting a facility-based audit process [19, 20]. Significant effort has been put into the process of conducting maternal and perinatal mortality audits with several studies investigating the underlying modifiable factors leading to these deaths in LMICs [21–23]. These modifiable factors were identified and pooled together in a systematic review conducted by Merali et al in 2014 [24]. The perinatal period, as defined by the WHO, “commences at 22 completed weeks (154 days) of gestation and ends seven completed days after birth” [25]. The perinatal period therefore covers stillbirths and the early neonatal period (first 7 days of life) for live newborns. The neonatal period commences at birth and ends at 28 completed days after birth [26]. However, the perinatal aspects of the mortality audits included in the systematic review were mostly restricted to stillbirths with minimal information on the avoidable gaps in the continuum of care of the small and sick newborn beyond the initial resuscitation after birth [27–39]. Based on this, we sought to pool together the modifiable factors reported in facility-based care of the newborn beyond the immediate care provided at birth with special interest in the care provided in the newborn units in LMICs. This was done through conducting a scoping review as it allowed us to examine literature of many types, did not restrict us to specific study designs, and allowed us to capture the diversity of work that may have been conducted in this field [40, 41].

The main objectives of this scoping review were: i) to identify the modifiable factors related to the care of newborns in LMICs from individual hospital audits ii) to assess the proportion of documented modifiable factors that are related to neonatal care in the newborn unit iii) to identify the most frequently occurring modifiable factors leading to adverse effects in the newborn units and iv) to assess the quality of the perinatal and newborn audit process in health facilities in LMICs to allow for the identification of modifiable factors and the recommendation and implementation of solutions that lead to change.

## Methods

The scoping review was conducted using the methodology outlined by Arksey and O’Malley and refined by Levac et al [42]. The components of this framework include: Identifying the research question, identifying relevant studies, study selection, charting data, collating, summarising and reporting results and consultation which is optional [43]. This approach was selected as the aim was to identify the breadth of the conceptualization of audit identified modifiable factors with respect to auditing the care provided to live newborns and to develop a newborn quality of audit process score [40]. Due to this, an assessment of the methodological limitations or risk of bias of the included studies is not required. We reported results using the PRISMA Extension for Scoping Reviews (PRISMA-ScR) guidelines [44].

### Search strategy

We conducted a search on literature published in the English language between 1965 and December 2019. The search was constructed on PubMed and adapted to other databases; CINAHL, EMBASE, LILACS, POPLINE and African Index Medicus. The literature search included the following combinations of exploded and focused MeSH headings and keyword searches; Perinatal, Maternal, Mothers, Neonatal, Newborn, Infant, Mortality, Audit, Clinical Audit, Death, Fatal Outcome, Avoidable, Preventable and Developing Countries. Bibliographic references from the selected studies were also searched for relevant articles on newborn mortality and clinical audits. Free-text searches on Google Scholar, snowballing from the reference sections of included papers and searching for literature from the South African PPIP were conducted to supplement the search.

#### PubMed search strategy

(“Mothers”[Mesh]) OR Maternal) OR “Infant, Newborn”[Mesh]) OR neonat*) OR perinat*)) AND (“Mortality”[Mesh]) OR “Death”[Mesh]) OR “Fatal Outcome”[Mesh]) OR Critical incident) OR clinical) AND audit) OR review) OR “Clinical Audit”[Mesh] AND (modifiable) OR preventable) OR avoidable) AND factors) OR gaps)

### Eligibility

Studies from LMICs were considered. The classification of the countries was determined using the World Bank criteria for stratification of countries based on the gross national income per capita [45]. The eligibility criteria are described on Table 1 above.

**Table 1:** Eligibility Criteria for Studies Included in the Scoping Review

Eligibility Criteria	
Inclusion Criteria	Exclusion Criteria
All study designs were eligible if the authors conducted hospital-based perinatal or neonatal clinical or mortality audits.	Audit studies that focused on deaths occurring in the community.
Audits conducted through the review of medical records, healthcare worker meetings and/or interviews of healthcare workers or patient families.	Reports and reviews that summarise the current state of research on the perinatal or newborn audit process.
Audit that identified at least one healthcare worker associated, and/or administrative associated modifiable factor in the care of a live newborn in the immediate post-natal period and following admission to the neonatal ward.	Studies that were exclusively focused on the antepartum and peripartum care of the mother.
Only literature published in the English language.	

All titles and abstracts of the identified articles were independently screened by two reviewers, MO and EM. Any disagreements that arose were settled by consultations between the two reviewers. When no consensus was reached, consultations were held with a third reviewer, JA. Full texts of potentially relevant papers were retrieved, read and subjected to the inclusion/exclusion criteria.

### Data extraction and synthesis

A data extraction tool was developed in consultation with the reviewers. This was adapted from the Joanna Briggs data extraction tool for scoping reviews and revised to include the variables that would answer the research question [46].

Information extracted included general study information such as authors, year of study, title of study, study design, country where the study was performed, context and study population. The data extraction tool shows this in more detail (See Additional file 1).

For quality assurance, full data extraction was performed independently by both MO and EM ensuring that each paper was read and extracted by at least two authors. Descriptive analysis of the data was used to capture the characteristics of the included studies. Findings from included studies were presented using a thematic approach based on the study objectives and a narrative analysis of each theme was conducted.

### Quality of audit process score

The audit process should observe certain standards as recommended by the World Health Organisation (WHO) for it to lead to meaningful change as a quality improvement strategy [12]. We identified seven crucial factors to ensure a successful audit process based on WHO recommendations: Presence of a multidisciplinary audit team (MDT), inclusion of health worker cadres involved in the care of the newborn in audit meetings to promote learning, use of a structured audit tool, regular structured meetings and completion of the audit cycle by categorisation of modifiable factors, generating action points from the identified modifiable factors, and implementing the recommendations [12, 12]. Based on these seven factors, a quality of audit process score was developed by MO in consultation with the 2nd and 3rd reviewers. The scoring method was designed to determine the quality of the audit process in individual studies to lead to meaningful change in the quality of care provided to the newborns [47].

The traffic light grading system was used to give a visual representation of the quality of the audit process in the individual studies [48]. Table 2 describes the methods used to score each of the seven factors. The audit processes in the included studies were scored independently by MO and EM and disagreements were resolved by consensus.

**Table 2:** Quality of Audit Process Score Adapted from WHO Recommendations for a Facility-Based Audit Process

Quality Process	Score 0	Score 1	Score 2
Presence of MDT	No MDT	Only clinicians	Other cadres
Presence of health workers in audit meetings	Only MDT	MDT and clinicians	MDT and other health workers
Frequent structured audit meetings	No meetings	• Not structured. • Held > 2-weekly.	Held at most 2-weekly.
Use of a structured audit tool.	No tool	Perinatal audit tool^a^	Neonatal audit tool.
Categorised modifiable factors	Not categorised	Phase delays^b^	Level of health system in which it occurs.
Recommendation of solutions	None	Not based on modifiable factors.	Based on modifiable factors.
Implementation of recommendations	None	Not based on recommendations^c^	Based on recommendations.

Abbreviations: MDT; Multidisciplinary audit team

^a^Perinatal audit tools that focus on stillbirths and the immediate care and resuscitation after birth and not on the continuum of newborn care beyond this period

^b^The 3-phase delay method refers to: 10 delays in seeking appropriate care 2) delays in reaching a health facility 3) delays in receiving appropriate care at a health facility

^c^Refers to the recommendations emanating from the audit meeting

## Results

Search Results.

**Fig. 1:**
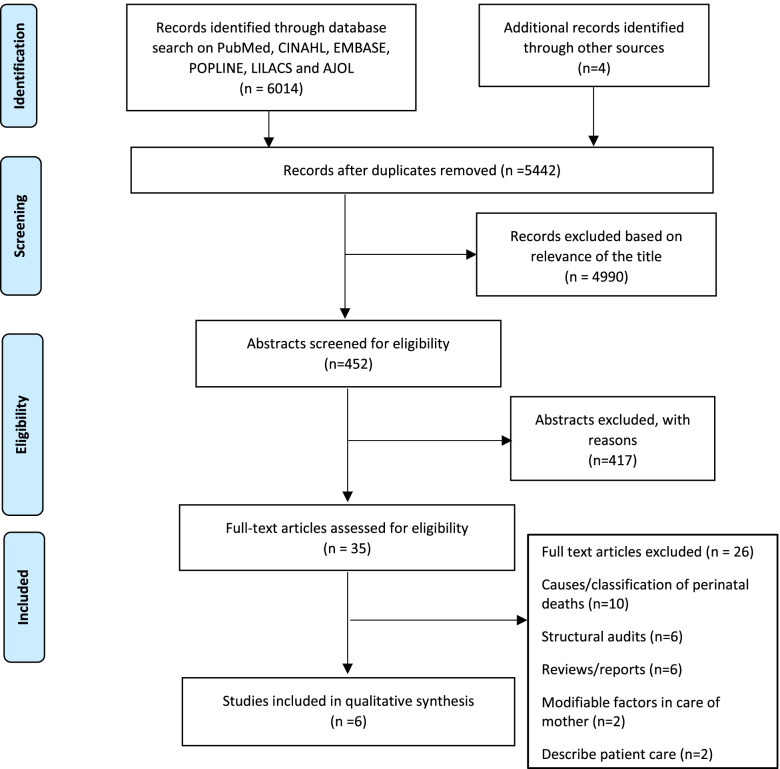
PRISMA Flow Chart on Literature Search Process

In total, we identified 6014 articles from database searches (Fig. 1). Four articles were identified from the reference lists of literature, a Google Scholar search and literature from the PPIP. After the screening of titles and abstracts, 35 articles were considered eligible for full-text screening. Reason for exclusion of 417 articles was; studies focused on maternal care or maternal deaths with no mention of newborn care, verbal autopsies, causes of perinatal or newborn death but not identification of modifiable factors, implementation of the audit process, structural audits, community based audits or reports, reviews or commentaries. Of these, three articles were not reviewed as they were inaccessible to us despite attempts to access them through various institutions; Oxford University and Vrije Universiteit. After review of the remaining 32 full text articles that were retrieved, we excluded 26. The main reasons for exclusion included: Articles that did not list down any modifiable factor related to the care provided to a newborn after birth, articles on causes of newborn mortality and articles that focused on the structure of health facilities rather than the process of newborn care (See Additional files 2, 3 and 4 for search results). The article selection process is presented in the PRISMA flow chart (Fig. 1).

### Data charting

Descriptive analysis of the data was used to capture the characteristics of the included studies as well as the causes of death.

#### Characteristics of included studies

A total of six studies met our inclusion criteria. Majority, (5/6) of the studies were from Africa, except one from Vietnam. Five of the six studies were conducted in public health facilities, and all included a tertiary level facility. Detailed descriptions of the included studies are provided in Table 3.

**Table 3:** Descriptive Characteristics of the Studies Included in the Scoping Review

Author and year	Country	Population	Definition of perinatal period	Type of audit	Methods	Setting	Number of cases audited	Facility based perinatal or neonatal mortality rates	% avoidable mortality in sample
**Demise et al. 2015** [49]	Ethiopia	Perinatal (Still births and early neonatal deaths)	Foetal deaths after ≥28 completed weeks of gestation and weighing ≥1000 g and live births dying within the 1st 7 days of life with a gestation of ≥28 completed weeks and a birth weight of ≥1000 g.	Mortality audit	Prospective clinical record review, staff and family interviews at a tertiary level public health facility	Maternity unit and newborn unit	61	49.8/1000 (PMR)	70%
**Musafili et al. 2017** [50]	Rwanda	Perinatal (Still births and early neonatal deaths)	Foetal deaths after ≥22 completed weeks of gestation and a weight of ≥500 g and live births dying within the 1st 7 days of life with a gestation of ≥22 completed weeks and a birth weight of ≥500 g.	Mortality audit	Prospective clinical record review, staff and family interviews at a secondary and tertiary level public health facility.	Maternity unit and newborn unit	250	32/1000 (PMR)	51%
**Nakibuuka et al. 2012** [51]	Uganda	Perinatal (Still births and early neonatal deaths)	Foetal deaths after ≥28 completed weeks of gestation and weighing ≥1000 g and live births dying within the 1st 7 days of life with a gestation of ≥28 completed weeks and a birth weight of ≥1000 g.	Mortality audit	Retrospective clinical record review at a private not for profit tertiary level health facility.	Maternity unit and newborn unit	120	52.8/1000 (PMR)	20%
**Kruse et al. 2014 **[52]	Vietnam	Neonatal		Mortality audit	Prospective clinical record review at a tertiary level health facility	Newborn unit	71	52/1000 (NMR)	23.50%
**Mbwele et al. 2012** [45]	Tanzania	Neonatal		Clinical audit	Prospective clinical record review at 1 tertiary level facility, 11 secondary level facilities and 2 primary level facilities.	Maternity unit, paediatric unit and newborn unit	82*		
**Okomo et al. 2015** [53]	The Gambia	Neonatal		Clinical audit	Retrospective clinical record review at a tertiary level public health facility	Newborn unit			

Abbreviations: NMR, Neonatal mortality rate; PMR, Perinatal mortality rate

The six reviewed articles included four studies that focused on perinatal or neonatal mortality audits, and two that were clinical audits. The clinical audits were of the processes of newborn care on life threatening cases or cases of patient deterioration that survive. The population included in the four mortality audit studies were majorly perinatal deaths (3/4) which occurred in the maternity units or newborn units. Demise et al and Nakibuuka et al had a similar definition for the perinatal period as described in Table 3. Only one mortality audit study exclusively focused on newborn deaths within the first 28 days of life occurring in the newborn unit. The population included in the clinical audits were newborns discharged alive from NBUs. In addition to newborns admitted to the NBU, Mbwele et al also focused on newborns in the maternity units and paediatric units [45, 53].

All included studies collected primary data and were either prospective (*n* = 4) or retrospective studies (*n* = 2). The mortality audit datasets comprised a total of 502 audited deaths, while the clinical audits comprised a total of 5367 process of care audits for newborns discharged alive. All the perinatal mortality audit studies included the facility specific perinatal mortality rates which ranged from 32/1000 to 52.8/1000 live births. One study, Nakibuuka et al included the difference in the hospital specific perinatal mortality rates at the beginning and end of the study; 52.8/1000 to 47.9/1000. One study, Kruse et al reported the neonatal mortality rate which was 52/1000 live births. All mortality audit studies included the percentage avoidable mortalities in the studies, and they ranged from 20 to 70%.

#### Causes of death

Five studies identified the causes of death within their newborn population. These were; four of the mortality audit studies and one of the clinical audit studies.

Eight causes of death were identified from the articles in this review. Three studies identified a single cause of death, while two studies included an immediate and underlying cause of death. The most described causes of death that were present in all studies were; complications of prematurity, intrapartum related events and severe neonatal infections. Severe infections were described as; meningitis, sepsis, pneumonia and peritonitis [52], while respiratory distress syndrome was the only described complication of prematurity [51]. The causes of death are summarised in Table 4 below.

**Table 4:** Causes of Death in Included Studies

Causes of neonatal deaths	Studies in which the cause of death was identified (***n*** = 5)
Complications of prematurity [49–52].	5
Intrapartum related events [49–51].	5
Severe infections [49, 50, 52].	5
Congenital malformations [49, 50, 52].	4
Neonatal jaundice [50].	1
Tetanus [54]	1
Haemorrhagic disease of the newborn [55].	1
Meconium Aspiration Syndrome [55].	1

The morbidities leading to hospitalisation of the newborns that were common in both clinical audit studies were: Possible severe bacterial infection, prematurity, perinatal asphyxia and neonatal jaundice.

The emerging themes from the data based on the study objectives included the modifiable factors in the process of care and the audit process in the facilities. A narrative analysis of each theme was conducted, and the results presented below.

#### Modifiable factors in newborn care

Only one study gave a definition of what was considered a modifiable factor. It was defined as ‘a risk factor that could have been avoided within the existing hospital context without the implementation of new technology or major organisational changes and if avoided in the actual clinical situation, the neonate would have been more likely to survive than die’ [52].

Two themes emerged in the narrative synthesis for the classification of modifiable factors:

1. Periods of newborn care.
2. Health system model in which newborn care occurs.

The periods of newborn care refer to the transition of care of a sick or small newborn from delivery up to the point of admission in the newborn unit. Three periods in the care of a newborn emerged from the narrative analysis of the data, these were: i) the period of immediate newborn care and resuscitation after birth, ii) the post-resuscitation care for the small and sick newborns and, iii) the period of care while in the newborn unit. The period of immediate care and resuscitation after birth was described as the care provided to the newborn shortly after birth. This includes drying, stimulation and resuscitation if required. The second period was described as the continued care provided to the small or sick newborn while still in the delivery room after the immediate resuscitation as they await to be transferred to the newborn unit. This also included the process of transfer to the NBU. The third period was the care provided to the newborn during and after admission to the newborn unit.

All the clinical audits fell under the last category as they only focused on newborns admitted to the wards or newborn units.

The four-level health system model includes i) individual patients, ii) health care providers, iii) infrastructure and resources and iv) environment [17]. The modifiable factor categories were adapted from this model and were defined as:

1. Health worker related factors which are factors related to the errors, oversights and deviations from accepted standards of care by the health workers involved in patient management [56].
2. Administrative factors which are factors whose resolution fell within the scope of the top-level hospital managers such as the hospital administrators and the hospital chief executive officers. These include modifiable factors related to i) financial, physical and human resources, ii) availability of medication, medical equipment, technology and materials and, iii) the political, policy and learning environment [56].
3. Patient oriented factors which are factors related to the interference by the caregivers in the clinical management of the newborns [56].

This method of categorising modifiable factors was also used in the systematic review by Merali et al. [24]

Overall, there were 31 modifiable factors in newborn care identified from both mortality and clinical audits. These have been categorised in Table 5 below based on the period of care and the level of the health system in which they occur.

**Table 5:** Identified Modifiable Factors in Newborn Care Categorised Based on Period of Care

**Period of immediate care and resuscitation after birth**^**a**^	
**Health worker factors**	Unsatisfactory preparation of neonatal resuscitation equipment [49]. Unsatisfactory preparation of medication e.g. surfactant [49]. Poor newborn resuscitation skills [49, 51]. Delayed initiation of resuscitation [50]. Poor communication between obstetrics staff and NICU team [49].
**Period of post-resuscitation care of the newborn**^b^	
**Health worker factors**	Insufficient prevention of hypothermia [49, 50]. Delay in transport to NICU [49].
**Period during care in the newborn unit**	
**Health worker factors**	Failure to provide adequate warmth [49]. Poor management of neonatal jaundice [50]. No RBS done on neonates with convulsions or reduced level of consciousness [45]. Neonates requiring oxygen not indicated to have received [45, 53]. Neonates requiring IV fluids not documented to have received [45]. Poor preterm feeding practices [49, 50]. Poor neonatal resuscitation [49, 50]. Irregular monitoring of vital signs [53, 50]. Delay in life saving interventions e.g. ET intubation due to poorly skilled health workers, blood transfusions [50]. Delayed recognition or response to danger signs [52]. Sub-optimal infection prevention measures [52]. Sub-optimal management of sepsis e.g. less aggressive antibiotic treatment or incorrect antibiotic dosing [45, 52]. No action on abnormal lab investigations – neonates who were HIV exposed did not receive prophylaxis [53]. Incomplete diagnosis – No indication of prematurity as a diagnosis [45]. Improbable diagnosis e.g. gastroenteritis in neonates [45]. Poor documentation of Apgar score [45]. Poor documentation of birth weight [45, 53]. Poor communication among health workers [49]. Sub-optimal internal transfers [52]. Delayed decision to referral [50].
**Administrative related factors**	Shortage of equipment e.g. monitors, airway devices & ventilators [45, 49, 50, 52]. Shortage of medication e.g. phenytoin [49, 50]. Shortage of staff [45, 52]. Inadequate laboratory capacity. Lack of capacity to perform bilirubin levels or blood cultures [45].
**Patient oriented factors**	Family perception of prognosis [52].

Abbreviations: ET, Endotracheal; HIV, Human Immunodeficiency Virus; IV, Intravenous; Lab, Laboratory; NICU, Neonatal intensive care unit; RBS, Random blood sugar

^a^No administrative and patient-oriented modifiable factors in period of immediate care and resuscitation

^b^No administrative and patient-oriented modifiable factors in post-resuscitation period

Majority of the modifiable factors 24/31 (77.42%) were identified during the care of the newborn in the newborn unit.

During the immediate care and resuscitation after birth and the period of stabilisation and transfer to the newborn unit, only health worker related modifiable factors were identified. Health worker related factors were also the majority in each period of newborn care.

#### Quality of perinatal and newborn audit process

A quality of care audit score was conducted for each study and the scores ranged from three to nine out of a maximum score of 14 (Table 6). The two clinical audit studies scored zero on all seven factors and are therefore not included in the table. The seven factors included the presence of a multidisciplinary audit team (MDT), inclusion of health worker cadres involved in the care of the newborn in audit meetings to promote learning, use of a structured audit tool, regular structured meetings, categorisation of modifiable factors, generating action points from the identified modifiable factors and implementing the recommendations emanating from the audit meeting.

**Table 6:**
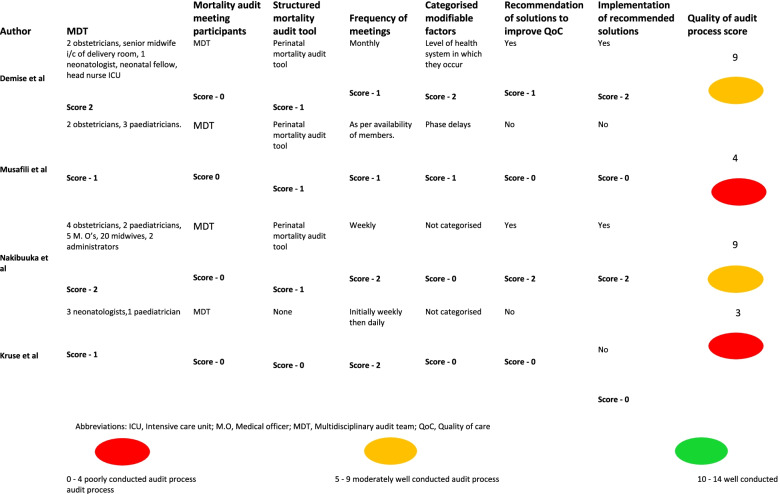
Quality of Audit Process Score in Included Mortality Audit Studies

For the four mortality audit studies, the categories with the highest scores were; the presence of an MDT where two studies each scored two points, the other two studies scored one point as the MDT only included clinicians. The frequency of audit meetings also scored highly with two studies having audit meetings at least two-weekly and the other two studies at least monthly.

Three of the four mortality audit studies used a structured perinatal audit tool therefore scoring one point each. One study did not use any structured audit tool.

Two mortality audit studies categorised the modifiable factors; one study categorised the modifiable factors according to the level of the health system and the other according to the phase delays. Two mortality audit studies made recommendations to solutions and implemented them.

The worst performing category was that of meeting participants where all scored zero points as they only included the MDT and not the rest of the clinical team.

The total scores showed that none of the mortality audit studies had a well conducted audit. Two studies had a moderately well conducted audit, and two had a poorly conducted audit.

#### Audit process as a quality improvement intervention

Only two of the six included studies documented the quality improvement measures that were brought about by identifying modifiable gaps, recommending solutions and implementing the recommendations. These are documented on Table 7 below. Nakibuuka et al described a reduction in perinatal mortality rate from 52.8/1000 to 47.9/1000 based on the measures taken predominantly in improving maternal care [51]. The other studies did not document the outcomes of the audit process.

**Table 7:** Quality Improvement Measures Based on Audit Process

Study	Modifiable Factors in Newborn Care	Quality Improvement Measures
Nakibuuka et al. [51]	• Poor newborn resuscitation skills.	• Three monthly training of intern doctors and nurses on neonatal resuscitation. • Provision of appropriate size ambu-bags and masks to the labour wards and newborn units. • Display of neonatal resuscitation protocols in labour wards and newborn units. • Setting aside neonatal resuscitation areas in labour wards and newborn units complete with a flat table, firm baby mattress and source of warmth.
Demise et al. [49]	• Unsatisfactory preparation of neonatal resuscitation equipment. • Unsatisfactory preparation of medication e.g. surfactant. • Poor newborn resuscitation skills.	• Newborn resuscitation trainings for midwives and physicians with plans for frequent trainings.
	• Poor communication between obstetrics staff and NICU team. • Delay in transport to NICU.	• Improved interdepartmental communication with the NICU team committed to be involved in counseling, planning and management of all high risk deliveries.
	• Insufficient prevention of hypothermia	• Scaling up efforts on skin to skin care of newborns in the delivery room, use of cellophane wraps and transport incubators, use of radiant warmers in the NICU.

## Discussion

The aim of this study was to 1) Identify the modifiable factors affecting the quality of care provided in newborn units in LMICs and 2) Assess the quality of the audit process to identify modifiable gaps that will generate actionable solutions that lead to improved quality of care. To the best of my knowledge, this is the first scoping review of audit identified modifiable factors in newborn unit care. There have been very few studies that have focused on auditing the quality of care provided in newborn units in low resource settings. Several modifiable factors have been identified in the continuity of care of a high risk newborn from birth to care provided in the newborn unit.

In total, 31 modifiable factors related to newborn care were identified from the included studies. From the identified modifiable factors, three time periods were distinguished along the continuum of care of the newborn who requires extra care after birth. These time periods represented:

1. The immediate care and resuscitation after birth.
2. The period of post-resuscitation care for the small and sick newborn. (Represents the period during which the newborn awaited transfer to the newborn unit and the period of transfer to the newborn unit)
3. The period of care in the newborn unit.

Previous studies that have identified modifiable factors in the care of a small and sick newborn have not attempted to make this distinction. This includes countries that have well established systems for auditing the newborn process of care such as; South Africa with the PPIP and the Netherlands with the Groningen system [54, 55]. Identifying the time period in which the modifiable factors occur is important because safety measures put in each of these periods work together to improve both the short and long-term outcomes of the small and sick newborns. Half of newborn deaths occur within 72 h of life, with two thirds of these occurring within the 1st 24 h of life [57]. To provide a better chance of survival in the newborn units, it is critical that life-saving interventions that will prevent fatal complications such as hypothermia, hypoglycaemia, early onset sepsis and respiratory complications should be instituted immediately after birth and continued through the post stabilisation period [58].

Similar to maternal and perinatal audit studies in the systematic review by Merali et al, the identified modifiable factors were more broadly oriented, and lacked the granularity to define the specific gaps in the care process [24]. For example, ‘poor preterm feeding practices’ broadly describes that there are deficiencies in preterm feeding practices, but does not explain where exactly the gaps are; mode of feeding? Type of feeds used? Volume of feeds? Frequency of feeding? Categorisation of modifiable factors aids in uniformity and comparison of results both at a national and international level. There are different methods of categorisation that have been used; three delays model, categorisation based on the level of the health system as used for this scoping review and the Groningen-system that was developed in the Netherlands [16, 24, 50]. The two methods that have been used by the studies included in this scoping review are the three-phase delay method and the method of categorisation by levels of the health system. Categorising modifiable factors by the level of the health system has been identified as a more comprehensive method as it shows the exact level within the health system in which action can be taken [16]. However, the categories should be detailed and clearly defined with guidelines in place to enable the allocation of modifiable factors into the appropriate category. Broad categories such as health worker related factors can be further refined into sub-categories such as; lack of technical skills, lack of knowledge, presence and use of guidelines, poor communication and poor documentation. For this review, the lack of detail in documenting the relationship between the modifiable factors and the process of care in the individual studies made it difficult to further refine the categorisation of the modifiable factors. The Groningen system in the Netherlands has attempted this. It gives very detailed and clearly defined modifiable factors [16]. The Groningen system groups modifiable factors into nine categories each with three to seven sub-categories. The categories are based on the process of care and not on the levels of care e.g. medical practice is one of the categories and has two sub-categories; diagnosis and management plan. The sub-categories are also further categorised into three sections: 1) use of guidelines 2) content of guidelines and, 3) common practice. A classification system that is more refined allows for detailed investigations into the relation between the modifiable factors and the adverse events. This leads to proper allocation of modifiable factors into appropriate categories, allows for uniformity, enables comparison at national and international levels and helps to define more specific actions required to address the problem [59, 24].

The period of care in the newborn unit accounted for the highest proportion of modifiable factors in newborn care. This does not necessarily reflect that most modifiable factors happen in the newborn unit, but that the included studies focused on this period. In an effort to increase the number of facility-based deliveries in LMICs, strategies such as the introduction of universal health coverage (UHC) and free maternity services have been put in place [60, 61]. This has resulted in increased access to inpatient care for the small and sick newborns [61]. Despite this, there has been minimal reduction in the neonatal mortality rate in these countries [3]. The results from this scoping review provide further evidence that in an effort to improve the quality of newborn care, identifying modifiable gaps during the period of care in the newborn unit should be given as much attention as the period of immediate care and resuscitation after birth which has been the area of focus of many perinatal audit studies [24]. In SSA, structures have been put in place to audit the care provided during the perinatal period which extends to seven days after birth through the Maternal and Perinatal Death Surveillance and Response (MPDSR) approach [62]. However, the perinatal component of the MPDSR approach is silent on the continued care provided to these newborns beyond the delivery room [63]. It would be useful to design a neonatal audit tool that complements the MPDSR tool by strengthening the newborn component. This will act as a structured guide to audit the newborn continuum of care in its entirety, including the period of care in the newborn unit. Understanding the modifiable factors in newborn unit care will be beneficial in developing strategies to reduce newborn mortality.

The most common category of modifiable factors in newborn unit care were health worker related factors. This was replicated across all other time periods and is similar to the findings in the systematic review by Merali et al. [24] This may be taken to suggest that majority of the deaths in the studies could have been prevented if health care workers performed optimally. However, the performance of health care workers is largely influenced by factors within the wider health system that influence their service provision [59]. A literature review conducted by the Johns Hopkins Center for Communication Programs identified the factors that impact health care worker performance [64]. The broad factors identified were:

1. Knowledge and competency barriers. This refers to the health care workers lacking the necessary knowledge and skills to effectively provide quality care to the patients [65].
2. Structural and contextual barriers. These refer to weaknesses within the context in which the health care workers are expected to perform optimally [66]. These are usually beyond the control or influence of the health workers and include: workforce insufficiencies, weak internal systems, lack of protocols and standards, political environment, limited supplies and equipment and community influence [67, 68].
3. Attitudinal barriers. These refer to the attitudes, beliefs, values and norms of the health care workers [65]. The identified factors that influence the attitudes of the health workers towards their practice include cultural norms and beliefs of the patients, motivating factors such as adequate remuneration, opportunities for knowledge and career progression and family wellbeing [64].

These findings from the scoping review have significant value as they emphasize how critical health workers are to improve newborn quality of care. However, the presence of health workers in newborn units does not alone ensure the safety of newborns. Understanding how health workers are supported and inhibited by the larger health system will guide stakeholders in anticipating and responding to these factors and put in the necessary measures to ensure that health workers can function optimally.

We designed a quality of care audit process score based on the WHO recommendations for conducting an effective audit process. This seven-category process of care score aims to assess how effectively an audit process has been conducted to identify modifiable factors and improve quality of care. The reviewers considered these seven factors as the bare minimum, however there are other important factors such as maintaining a favourable environment during the audit meeting. This includes; a no-name, no blame, non-judgemental environment, maintaining confidentiality, an environment that encourages equal participation among cadres and an environment that promotes learning [12]. These have not been included in the quality of audit process score as they cannot be objectively quantified.

A multidisciplinary audit committee (MDT) consisting of representatives from the different cadres responsible for care of the newborns is essential. This instils a systems approach in understanding the roles of the different departments in improving the quality of newborn care [12]. The presence of an audit committee enforces accountability from the hospital managers and the care providers by enforcing implementation of action plans. The size and composition of the audit committee is however highly dependent on the staffing of the hospital. Nyamtema et al*,* [68] identified the absence of audit committees as a barrier to a successful audit process in Tanzania. For this reason, we gave the highest score to the studies that had more than one cadre represented in the MDT.

The audit meeting should have a strong educational aspect as the health workers should learn from the modifiable gaps that led to the poor outcome in the patients preventing similar mistakes in other patients. This therefore means that audit meetings should be attended not only by members of the MDT but should reflect the frontline newborn care in the hospital [12]. With more diverse attendance, there will be richer discussions around patient care with equal focus on all aspects of the health system. This will also enforce implementation of recommended solutions by different departments. This is therefore the reason we gave the highest score to the meetings that had a more diverse attendance.

In LMICs where neonatal mortalities are high, it is recommended that mortality and morbidity audit meetings be held frequently; weekly or bi-weekly. This allows the health workers to audit as many events as possible. It is also recommended that the timeframe from an event to its discussion in a mortality or morbidity meeting should be at least a week. This is to ensure that the events are still fresh in the minds of the health workers to have a more meaningful discussion [12, 47]. In South Africa where the process of perinatal audits has been the most successful in SSA, a study conducted by Belizán et al [67] identified the frequent audit meetings as a factor for their success. The hospitals have daily or weekly meetings at the NBU level, monthly meetings at the hospital level and annual meetings at the regional level [67]. Sandakabatu et al, [69] from Solomon Islands identified weekly perinatal audit meetings as a means to capture as many perinatal deaths as possible in a high mortality setting. We therefore gave the highest score to the studies that held meetings less than monthly.

A structured audit tool provides a systematic guide to document the care provided to newborns throughout the continuum of care. The design of the audit tool is based on a root cause analysis method, this makes it possible to identify the modifiable factors leading to unfavourable events at different stages in the process of care. The root cause of the problems can be identified by structuring the audit tool to answer the “5 Why Questions” or the “6 What Questions” [70, 71].

Structured audit tools used for newborn audits in LMICs have been identified. These audit tools include:

1. The WHO Stillbirth and Neonatal Death Case Review Form.
2. WHO Child and Neonatal Death Review Form.
3. Kenyan Ministry of Health (MOH) Maternal and Perinatal Death Surveillance and Response Tool.
4. PPIP tools from South Africa [12, 47, 63, 72].

A common feature in all these tools is that they focus on the care provided to the mother during the peri-partum period and the immediate resuscitation of the newborn after delivery. They provide no capacity to audit the care provided to the small and sick newborns admitted in the newborn units. For this to be done effectively, a newborn unit audit tool is required. For this reason, we gave a score of one to studies that used a perinatal audit tool, and a score of two for the use of a newborn audit tool that focuses on the continuum of newborn care across all periods. One study from Rwanda that did not meet the inclusion criteria for the scoping review reports to have used a newborn audit tool [73]. However, despite several attempts, we have been unable to gain access to the tool.^1^ After a careful search through the relevant literature, we therefore believe that there is no newborn unit audit tool for use in LMICs.

The recommendation of solutions arising from the action points generated during an audit meeting, and implementation of the recommendations is an important aspect of an audit process. Kongnyuy et al [68 and Nyamtema et al [68 identified that an audit process is meaningful when solutions are recommended and implemented to prevent the same modifiable factors from re-occurring. Failure to do this leads to lack of confidence in the audit process by the hospital staff as there will be no changes arising from the audit process [74]. The scope of the included papers was to identify the modifiable gaps in newborn care, and not necessarily to report on the actions taken on the identified gaps. The authors of the included studies may have therefore opted not to report on the recommendations made from the identified modifiable factors or the implementation of recommendations. This may have led to a lower quality of audit process score than required. The quality of audit process score provides a framework to guide on the reporting of the audit process in future studies.

Quality improvement is defined as “better patient experience and outcomes achieved through changing provider behaviour and organisation through using a systematic change method and strategies.” [75] The effectiveness of the audit process as a quality improvement initiative is dependent on its ability to measure the quality of patient care as a whole and along the continuum of care. This holistic approach in viewing system performance allows for multifaceted interventions engaging the three levels; micro level (health care provider and patient level), meso level (hospital and district levels) and macro levels (national level) [76]. This ensures sustained change in the health system with the ultimate goal being a reduction in mortality rate. Only one of the included studies by Nakibuuka et al documented an outcome from the audit process. They reported that the perinatal mortality rate reduced from 52.8 to 47.9 per 1000 live births within the one-year study period. They attributed this reduction in mortality to the training of staff and the purchasing of essential equipment that came as a result of recommendations from the mortality audits [51]. A report by the South Africa Every Death Counts Writing Group reported outcomes of the Child and Perinatal PIP from South Africa. The child audit programme resulted in a 60% decrease in mortality due to Pneumocystis Jirovecii Pneumonia deaths and a 37% reduction in inpatient deaths. This was due to an increase in use of Highly Active Antiretroviral Therapy (HAART) based on recommendations emanating from the audit process. The perinatal audit programme resulted in widespread upscaling of Kangaroo Mother Care (KMC) for preterm newborns and this resulted in a 30% reduction in neonatal mortality in the hospitals that implemented KMC. The report however reported that despite efforts to improve use of the partograph this was not successful in many of the hospitals. This was attributed to factors within the system such as shortage of staff and high staff turnover. This may portray the challenges experienced in bringing about change when the actions are required from a higher administrative level [18]. A study conducted by Gaunt et al in South Africa over a three-year period after the introduction of the PPIP showed that there was an improvement in care to the pregnant mothers. One of the major successes they reported was in encouraging pregnant women to get a Human Immunodeficiency Virus (HIV) test. This was done through working with the antenatal clinic teams to encourage mothers to get tested and offering dual Prevention of Mother to Child Transmission and Testing (PMTCT) treatment to patients who tested positive. By 2008, 99.8% of women delivering in their facility knew their HIV status. The study also reported a reduction in the six monthly PMR (49.1–22.4/1000 live births) between 2005 and 2008 despite an increase in the number of deliveries at the facility through the study period. There may have been some influence from other interventions on the positive outcomes from the study [77]. Other studies have reported a reduction in PMR as a long-term outcome of the audit process [78, 79]. The high-income and LMICs that have well established quality of care audit processes report significant changes in the processes of patient care. This is evidence that the audit process does lead to an improvement in quality of care.

The strengths of the study are that the search strategy used was iterative as the search terms were redefined as the researchers got more familiar with the literature. This made it adequate to capture a wide scope of papers. We also followed all five steps of the modified Arksey and O’Malley framework for scoping reviews. The limitations were that: 1) there were few numbers of papers that met the inclusion criteria and therefore limited data on the modifiable factors on facility-based newborn care 2) the selection of papers was based on identification of modifiable factors and therefore the studies did not capture the quality improvement effects of the audit process. We did not include this as part of our objectives but however recognize the importance of discussing the quality improvement effects of the audit process.

## Conclusion

The findings from this review suggest that understanding and categorising the modifiable factors in newborn care is an effective strategy to identify the levels of the health system at which action can be taken. The method of categorisation by levels of the health system is an effective method, however, it should be refined further to allow for more specificity into where exactly the problem lies. This will allow the health workers to define more specific actions to prevent the modifiable gaps from recurring.

An audit tool that is specifically designed to audit newborn care in LMICs is required. This will enable health workers to systematically identify the modifiable gaps in newborn unit care from admission to the event of interest.

The newborn audit process in LMICs should be standardised. The seven factors in the quality of audit process score provide a framework for the design of a standard operating procedure for the conduct of the newborn audit process. This is a useful process to maintain the standards of audit processes in the health facilities.

## Supplementary Information

**Additional file 1.** Scoping review data extraction tool.

**Additional file 2.**

**Additional file 3.**

**Additional file 4.**

## Data Availability

The datasets used and analysed from the current study are available from the corresponding author on reasonable request.

## Abbreviations

HICs: High Income Countries
LMICs: Low and low-middle income countries
MOH: Ministry of Health
MDT: Multidisciplinary audit committee
NBU: Newborn Unit
NMR: Neonatal Mortality Rate
PPIP: Perinatal Problem Identification Programme
SSA: Sub Saharan Africa
UHC: Universal Health Coverage
WHO: World Health Organisation

## Footnotes

1 Several attempts have been made to contact the authors with no success.
